# Editorial: Diabetes and mental health: from understanding biomedical and social determinants, to promoting wellness in diabetes

**DOI:** 10.3389/fendo.2024.1517615

**Published:** 2024-11-15

**Authors:** Martine Elbejjani, Sonya S. Deschênes, Hala Ahmadieh, Mona P. Nasrallah

**Affiliations:** ^1^ Clinical Research Institute, Faculty of Medicine, American University of Beirut, Beirut, Lebanon; ^2^ Department of Internal Medicine, Faculty of Medicine, American University of Beirut, Beirut, Lebanon; ^3^ School of Psychology, University College Dublin, Dublin, Ireland; ^4^ Healthplus Diabetes and Endocrinology Center, Department of Endocrinology, Khalifa University, Abu Dhabi, United Arab Emirates; ^5^ Division of Endocrinology and Metabolism, Department of Internal Medicine, Faculty of Medicine, American University of Beirut Medical Center, Beirut, Lebanon

**Keywords:** depression, diabetes mellitus, mental health, anxiety, diabetes - quality of life

Diabetes and mental health disorders are health priorities worldwide, with mounting evidence demonstrating their intertwined connection. In particular, the association between diabetes and depression is frequently reported. Both conditions are highly prevalent, have been on the rise globally, and are main causes of morbidity and mortality (1, 2). Data from clinical and community studies consistently report a complex co-morbidity between diabetes and depression; people with depression have higher rates of type 2 diabetes (3), and conversely, people with diabetes are found to be twice more likely to have depression (4).

The 13 articles included in this Research Topic highlight novel and different facets of the emergence, progression, and outcomes of the diabetes-mental health connection. The articles span investigations of both hypothesized temporal relationships of this co-morbidity, its consequences on management, complications, and well-being, as well as its intersection with other disorders and risk factors.

The work of Sanchez-Carro et al., Huang et al., and Mishra et al., examines the potential role of depression and psychosocial factors in influencing the risk for diabetes and dysglycemia. Investigating metabolic disease development in a prospective Greek cohort (n=755), Sanchez-Carro et al. found that participants with both depression and anxiety had more pronounced inflammation profiles at baseline, and that participants with depression had a higher risk for developing diabetes over the next ten years. Mishra et al. reported that participants with more severe depressive symptoms had higher glycemic variability in a single-center pilot study involving flash glucose monitoring in individuals with depression and without diabetes. Huang et al., examined diabetes trends in the US from 2005 to 2018 and the contribution of 31 modifiable and non-modifiable risk factors using data from the National Health and Nutrition Examination Survey. Their findings showed increases in diabetes prevalence from 12.2% in 2005-2006 to 17.1% in 2017-2018 and that changes in biological, demographic, anthropometric, psychosocial, and genetic domains accounted for these increasing trends by 46.2%, 41.5%, 35.3%, 21.3%, and 17.3%, respectively. These findings from diverse populations and settings suggest that adverse mental health experiences are associated with higher risk for diabetes and glycemic variability; they also underline that this association spans multiple mental health conditions, psychosocial factors, and health indicators.

In parallel, five studies published in this Research Topic are in support of the hypothesis that diabetes is related to the occurrence of mental health conditions. The cross-sectional study by Yadav et al. on 1125 emerging adults with diabetes, as part of the National Health and Nutrition Examination Survey, found that a major depressive episode was more likely to occur among ethnic minorities and among women. Depression and anxiety were also more common in certain specific settings such as the post-partum period, as demonstrated by Zeng et al. in a sample of 406 women with or without gestational diabetes (GDM). The women with GDM reported higher anxiety 42 days after delivery in a self-reported questionnaire. Similar associations were reported in a meta-analysis by Jin et al., which included 10 studies on women with GDM. Depression was more common among women with GDM as compared to those without GDM across multiple countries and in prospective and retrospective study designs. Furthermore, it was more common in low- and middle-income countries, as compared with high income countries. Together, these findings highlight that depression and anxiety are more prevalent with diabetes among minorities, women, lower socioeconomic status, and specific settings, and underscore that common determinants between both conditions can be psychosocial and socioeconomic/environmental in nature. Other articles suggest that physiologic processes may also play a role. Chamseddine et al., found different associations between mental health and fasting blood glucose (FBG) and hemoglobin A1c (HbA1c) levels depending on the range examined, with only increases in levels in the range consistent with diabetes (≥126 mg/dl and ≥6.5%, respectively) showing patterns of associations with higher depression and anxiety symptoms. This suggests a differential shift in mental health risk in the clinical spectrum of glycemic indicators. In a systematic review and meta-analysis by Shea et al., pooling 31 studies of 2.1 million adults with nonalcoholic fatty liver disease (NAFLD), the presence of depression and anxiety was 26.3% and 37.2% more likely to occur, respectively. NAFLD is a silent clinical condition closely associated with type 2 diabetes and shares its common risk factors such as inflammation, insulin resistance, and genetic predisposition. These findings highlight the importance of early detection and better characterization of processes underlying the transition towards diabetes development and its implications for mental health. Further investigating the co-occurrence of mental health symptoms in the context of diabetes, Zhang et al., present cross-sectional networks of depressive and anxiety symptoms among 1,685 older adults with diabetes (unspecified type) from the 2017–2018 wave of the Chinese Longitudinal Healthy Longevity Survey (CLHLS). They found that “Feeling blue/depressed”, “Nervousness or anxiety”, “Uncontrollable worry”, “Trouble relaxing”, and “Worry too much” were the most central symptoms and might therefore contribute most to the development and maintenance of depression and anxiety, and that symptoms related to “Nervousness or anxiety” and “Everything was an effort” were the strongest nodes to bridge together symptoms of anxiety with symptoms of depression.

In addition to improving our understanding of their emergence and temporal link, data on the progression and implications of diabetes and mental health co-morbidities are critical to guide informed treatment and management strategies. The studies by Yeung et al. and Schmitz et al. investigate downstream complications of this co-morbidity. Using 6-year prospective data from the Hong Kong Diabetes Register (2013–2019), Yeung et al., found that elevated depressive symptoms were associated with incident cardiovascular disease, ischemic heart disease, and all-cause mortality in 4525 Chinese patients with diabetes, accounting for health-related quality of life and self-care factors. The study by Schmitz et al., reported an interaction between depression and high ultra-processed food consumptions in a prospective cohort of middle-aged adults with type 2 diabetes, wherein participants with a combination of elevated depressive symptoms or antidepressant use and high ultra-processed food consumption were at higher risk of new-onset diabetes-related microvascular and macrovascular complications. Both studies document associations of high magnitude (over double the risk of complications), underlining the potentially severe and important sequela of depression-diabetes co-morbidity and its interplay with daily health-related behaviors and other disease processes.

This Research Topic also includes work that examines instruments for assessing psychological barriers to treatment and the impact of newer diabetes management technologies. Improving tools assessing psychological barriers to treatment and diabetes-related distress, depression, and other mental health conditions, and adapting them to different populations and settings, is instrumental for better patient care. The development and psychometric assessment of the Chinese Barriers to Insulin Treatment Questionnaire (BIT-C) by Ma et al. is a good example of the importance of such efforts. The associations between progress in diabetes technology and improvements in mental health and wellbeing was demonstrated by Cyranka et al. who reported improvements in the quality of life and wellbeing of 18 adults with type 1 diabetes approximately one year following a switch from multiple daily injections and self-monitoring of blood glucose to the advanced hybrid closed-loop system. Participants were previously naïve to modern diabetes technologies and improvements included increases in life satisfaction, self-esteem, and well-being, and self-efficacy, and decreases in anxiety.

In conclusion, the articles included in this Research Topic are a collection of efforts from around the world to better understand the diabetes-mental health connection, its underlying causal pathways, manifestations, and implications for health and treatment outcomes. Together, the articles put forward future research opportunities and directions emphasizing the value of investigating each of these conditions’ building blocks and trajectories, with more diversified and at-risk populations, and their interplay with sociodemographic, biological, and health-related factors.
